# Chromosome Dynamics Visualized with an Anti-Centromeric Histone H3 Antibody in *Allium*

**DOI:** 10.1371/journal.pone.0051315

**Published:** 2012-12-07

**Authors:** Kiyotaka Nagaki, Maki Yamamoto, Naoki Yamaji, Yasuhiko Mukai, Minoru Murata

**Affiliations:** 1 Institute of Plant Science and Resources, Okayama University, Kurashiki, Japan; 2 Department of Rehabilitation Sciences, Kansai University of Welfare Sciences, Kashiwara, Japan; 3 Division of Natural Science, Osaka Kyoiku University, Kashiwara, Japan; Virginia Tech, United States of America

## Abstract

Due to the ease with which chromosomes can be observed, the *Allium* species, and onion in particular, have been familiar materials employed in cytogenetic experiments in biology. In this study, centromeric histone H3 (CENH3)-coding cDNAs were identified in four *Allium* species (onion, welsh onion, garlic and garlic chives) and cloned. Anti-CENH3 antibody was then raised against a deduced amino acid sequence of CENH3 of welsh onion. The antibody recognized all CENH3 orthologs of the *Allium* species tested. Immunostaining with the antibody enabled clear visualization of chromosome behavior during mitosis in the species. Furthermore, three-dimensional (3D) observation of mitotic cell division was achieved by subjecting root sections to immunohistochemical techniques. The 3D dynamics of the cells and position of cell-cycle marker proteins (CENH3 and α-tubulin) were clearly revealed by immunohistochemical staining with the antibodies. The immunohistochemical analysis made it possible to establish an overview of the location of dividing cells in the root tissues. This breakthrough in technique, in addition to the two centromeric DNA sequences isolated from welsh onion by chromatin immuno-precipitation using the antibody, should lead to a better understanding of plant cell division. A phylogenetic analysis of *Allium* CENH3s together with the previously reported plant CENH3s showed two separate clades for monocot species tested. One clade was made from CENH3s of the *Allium* species with those of Poaceae species, and the other from CENH3s of a holocentric species (*Luzula nivea*). These data may imply functional differences of CENH3s between holocentric and monocentric species. Centromeric localization of DNA sequences isolated from welsh onion by chromatin immuno-precipitation (ChIP) using the antibody was confirmed by fluorescence *in situ* hybridization and ChIP-quantitative PCR.

## Introduction

*Allium* species including onion (*A. cepa*), welsh onion (*A. fistulosum*), garlic (*A. sativum*) and garlic chives (*A. tuberosum*) have been used as foods and/or spices around the world. Since most of the species have large-sized chromosomes and high mitotic indices in root-tips, they, and onion in particular, have also been used as material for cytogenetic experiments in biology as well as for monitoring mitotic toxicity [1]. However, given the lack of molecular markers on the centromeres, information concerning cell division is limited in comparison with that of animal cells, not only for the species analyzed here, but also for other plant species possessing large chromosomes [2]–[4].

Investigation of *Allium* chromosomes at the molecular level has revealed some special features; the species lack typical *Arabidopsis*-type telomere sequences, and their chromosome ends are maintained with subtelomeric tandem repeats [5]. A number of different tandem repeats have been identified in subtelomeric heterochromatins which may relate to function [6]–[11]. However, other chromosomal regions including centromeres have yet to be analyzed.

Centromeres consist of specific DNA and form a functional complex, the kinetochore, with specific proteins utilized for the equal distribution of chromatids to daughter cells at mitosis and meiosis [12]. Centromere-specific histone H3 (CENH3) is one of the most fundamental centromeric proteins involved in recruiting other centromeric proteins [13]. CENH3 was first identified as CENP-A in humans [14], and subsequently found in a large number of plant species including Brassicaceae, Solanaceae, Leguminosae, Poaceae and Juncaceae species [15]–[25]. These analyses revealed conserved histone fold domains and variable amino-tails uniqueness of CENH3s, and that Loop 1 in the histone fold domains is important for centromeric localization of CENH3s. Most investigations of CENH3 have utilized monocots sampled from cereal crops belonging to Poaceae, such as maize [24], rice [15], sugarcane [17] and barley [19], although *Luzula* species, belonging to Juncaceae, have also been investigated given their special holocentric feature [16], [26], [27]. This makes it difficult to evaluate the difference between the holocentric CENH3 and other monocot CENH3.

Since CENH3 comprises part of the core histone that binds directly to DNA at centromeres, centromeric DNA has been isolated from several plant species by chromatin immuno-precipitation (ChIP) using antibodies against CENH3 [15], [17], [18], [21], [22], [24], [25], [28]–[31]. Those results revealed that centromeric DNA is species-specific, comprising tandem repeats of several hundred bp units and/or centromere-specific retrotransposons. For example, all centromeres of rice consist of a 156-bp tandem repeats and a gypsy-type retrotransposon [15]. In higher eukaryotes, one type of tandem repeats is located on all centromeres. However, chromosome-specific and centromeric tandem repeats were identified in chicken and tobacco [32], [33]. Recently, similar chromosome-specific distribution of centromeric tandem repeats was also reported in potato [34].

In human cells, fluorescent protein fusions with CENH3 (CENP-A) and other proteins have been utilized to visualize microtubules, chromatin, centromere and nuclear envelope, and to study the dynamics of cell divisions [35]. In plant, tobacco BY-2 cells expressing GFP-fused tubulin were produced to analyze cell division, resulting in identification of a plant-specific structure, the preprophase band (PPB) [36]–[38]. PPB is present from preprophase to prophase at the at a site corresponding to the future position of the metaphase plate. Abnormal PPB was found to induce abnormal cell divisions [37]. Similar cell dynamics was also visualized by GFP-fused proteins in *Arabidopsis* [39]. However, the information from these plant experiments is limited, because information about the position and movement of the centromeres was not provided.

In this study, we identified cDNAs encoding CENH3s in four *Allium* species including onion (*A. cepa*), welsh onion (*A. fistulosum*), garlic (*A. sativum*) and garlic chives (*A. tuberosum*), and the phylogenetic analysis including amino acid sequences of previously reported plant CENH3s revealed uniqueness of *Luzula* CENH3s. A polyclonal-antibody was raised against a peptide sequence deduced from the CENH3 amino acid sequence of welsh onion, and the antibody recognized the kinetochores on chromosomes in all four *Allium* species by immunostaining. Moreover, immunohistochemical staining of onion root sections using the antibody allowed clear visualization of the three-dimensional (3D) cellular organization and location of dividing cells in roots. The antibody was also used in a ChIP experiment to isolate centromeric DNA from welsh onion. Centromeric localization and co-localization with CENH3 of the centromeric DNA isolated were confirmed by fluorescence *in situ* hybridization (FISH) and real-time quantitative PCR (qPCR).

## Results

### Identification and Characterization of Centromere-specific Histone H3 Variants in *Allium* Species

**Figure 1:**
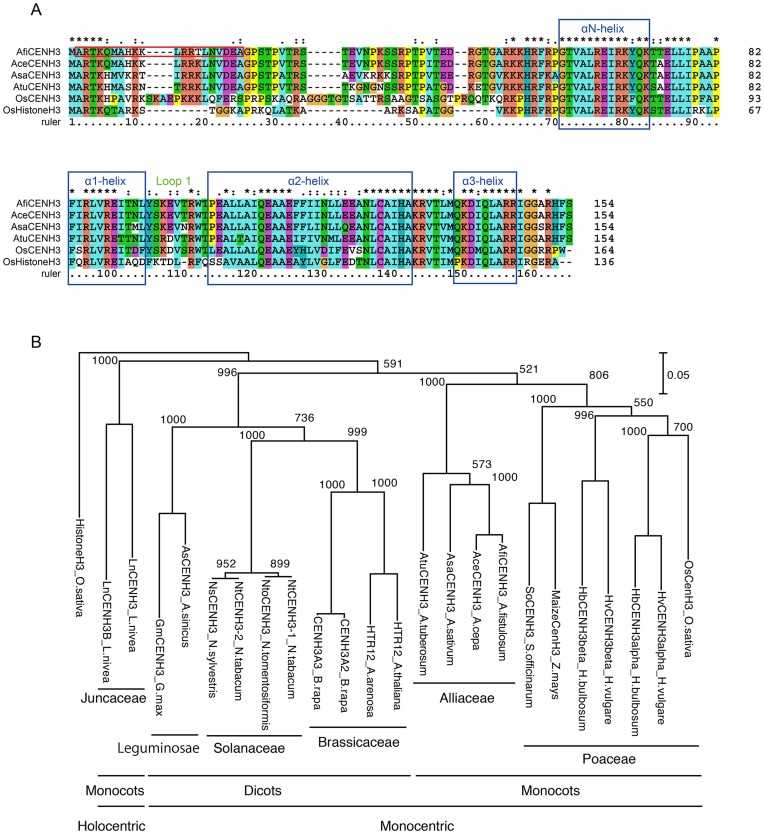
Alignment and phylogenetic tree of CENH3s. (A) Amino acid sequence alignment of CENH3s of *A. fistulosum* (AfiCENH3), *A. cepa* (AceCENH3), *A. sativum* (AsaCENH3), *A. tuberosum* (AtuCENH3) and *Oryza sativa* (OsCENH3), and a canonical histone H3 of *O. sativa* (OsHistoneH3). Identical, similar and weakly similar amino acids are indicated by asterisks, colons and dots, respectively. A red box indicates the amino acid residues used for the generation of antibody against AfiCENH3. Blue boxes correspond to helices in a histone fold domain. (B) Phylogenetic tree based on CENH3 amino acid sequences of plant species. A canonical histone H3 from *O. sativa* was used as an outgroup. Bootstrap values in 1000 tests are indicated on the branches.

We searched for onion CENH3 in an onion expressed sequence tag (EST) database of the gene index project using the tblastn program with the amino acid sequence of OsCENH3 [15] as a query. One EST group (CF445328) showed 66% sequence similarity to the query sequence. To obtain full-length cDNA of the gene, we conducted a rapid amplification of cDNA ends (RACE) experiment using onion root cDNA as a template, and successfully amplified AceCENH3 (GenBank accession number AB600275). AceCENH3 comprises a 462 bp ORF that codes for 154 amino acids (Figure 1A). Similarly, we successfully isolated cDNA of the orthologous genes from seedlings of *A. fistulosum* and *A. tuberosum* and from roots of *A. sativum*, each of which putatively codes for 154 amino acids (Figure 1A). These CENH3s were named AfiCENH3 (*A. fistulosum*: GenBank accession number AB571555), AtuCENH3 (*A. tuberosum*: GenBank accession number AB571557), and AsaCENH3 (*A. sativum*: GenBank accession number AB571556).

The amino acid sequences deduced from the ORFs were aligned with those of rice CENH3 (OsCENH3) and canonical histone H3 (Figure 1A). Although the amino acid sequences of the four *Allium* CENH3s are similar, some species-specific variations were found. Compared with the amino acid sequence of AceCENH3, AfiCENH3 shows 98% identity, whereas AtuCENH3 and AsaCENH3 each show 85% identity. The *Allium* CENH3s possess longer Loop1 domains than canonical histone H3, as found in other eukaryotes [40].

Phylogenetic analyses using the amino acid sequences of the CENH3s revealed the phylogenetic and functional relationships of the proteins (Figure 1B). CENH3s of the *Allium* species (Alliaceae) formed a clade in the phylogenetic tree. The Alliaceae clade was close to the Poaceae clade, both of which belong to the Monocots clade. Although *Luzula nivea* is a monocot species, it formed an independent clade (Figure 1B), perhaps due to the special feature of the CENH3s in the holocentric species.

**Figure 2:**
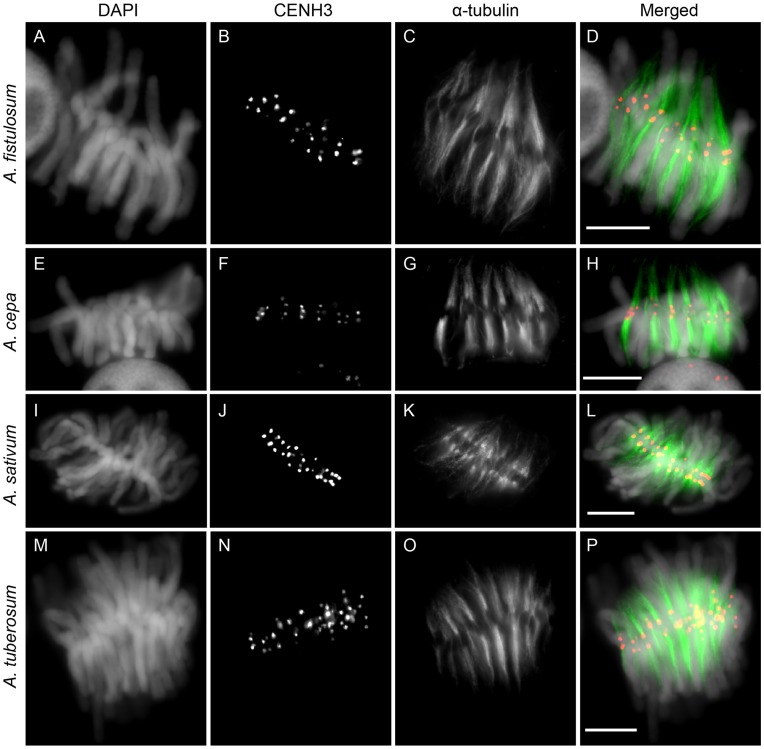
Immunostaining of chromosomes of *Allium* species using an anti-AfiCENH3 antibody. (A), (E), (I) and (M): DAPI stained chromosomes. (B), (F), (J) and (N): Immunosignals of an anti-AfiCENH3 antibody. (C), (G), (K) and (O): Immunosignals of an anti-α-tubulin antibody. (D): Merged image of (A–C). (H): Merged image of (E–G). (L): Merged image of (I–K). (P): Merged image of (M–O). (A–D): *A. fistulosum.* (E–H): *A. cepa.* (I–L): *A. sativum*. (M–P): *A. tuberosum.* Scale bar, 10 µm.

An anti-AfiCENH3 antibody was raised against a synthetic peptide comprising N-terminal amino acid residues 2–21 based on the AfiCENH3 deduced amino acid sequences (Figure 1A), and was used to determine the chromosomal location of the protein in the *Allium* species (Figure 2). Immunosignals obtained when using the anti-AfiCENH3 antibody appeared preferentially on the centromeres and localized at the tip of microtubule bundles visualized by alpha-tubulin staining not only in *A. fistulosum* but also in the *Allium* species *A. fistulosum, A. tuberosum* and *A. sativum* (Figure 2 and S1). This result indicates that AfiCENH3 identified in this study is an authentic CENH3 in *A. fistulosum,* and that the anti-AfiCENH3 antibody generated can cross-react with CENH3 orthologs of other *Allium* species.

### Immunohistochemical Staining of Root Sections

**Figure 3:**
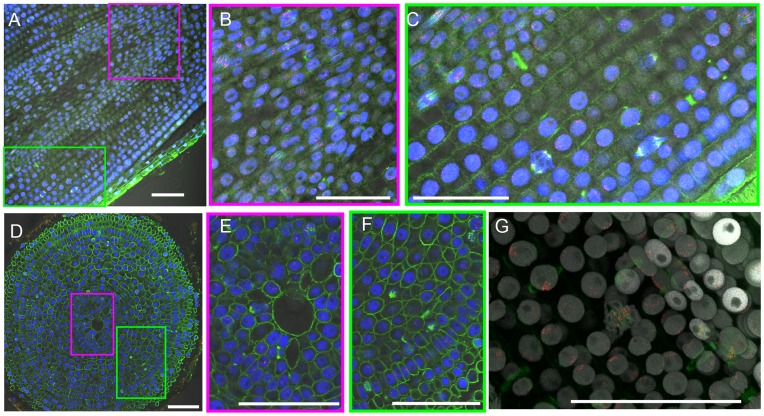
Immunohistochemical staining of onion root sections. (A) Vertical slice of an onion root. The magenta and green boxes indicate columella and cortex, respectively. Anti-AfiCENH3, anti-α-tubulin and DAPI signals are indicated in red, green and blue, respectively. (B) and (C) are close-up images of the magenta and green boxes in (A), respectively. (D) Horizontal slice of an onion root. Anti-AfiCENH3, anti-α-tubulin and DAPI signals are indicated in red, green and blue, respectively. The magenta and green boxes indicate columella and cortex, respectively. (E) and (F) are close-up images of the magenta and green boxes in (D), respectively. (G) A 3D image constructed from a set of Z-stack pictures. Anti-AfiCENH3, anti-α-tubulin and DAPI signals are indicated in red, green and grey, respectively. Scale bar, 100 µm.

In addition to the immunostaining of squash preparations, immunosignals were obtained when using the anti-AfiCENH3 and anti-α-tubulin antibodies in root sections. This analysis assisted in establishing the overall location and direction of dividing cells *in situ*. Signals appeared frequently in dividing columella cells and the cortex (Figure 3A–F), and vertical cell divisions were found to occur more frequently than horizontal cell divisions (Figure 3A–F).

**Figure 4:**
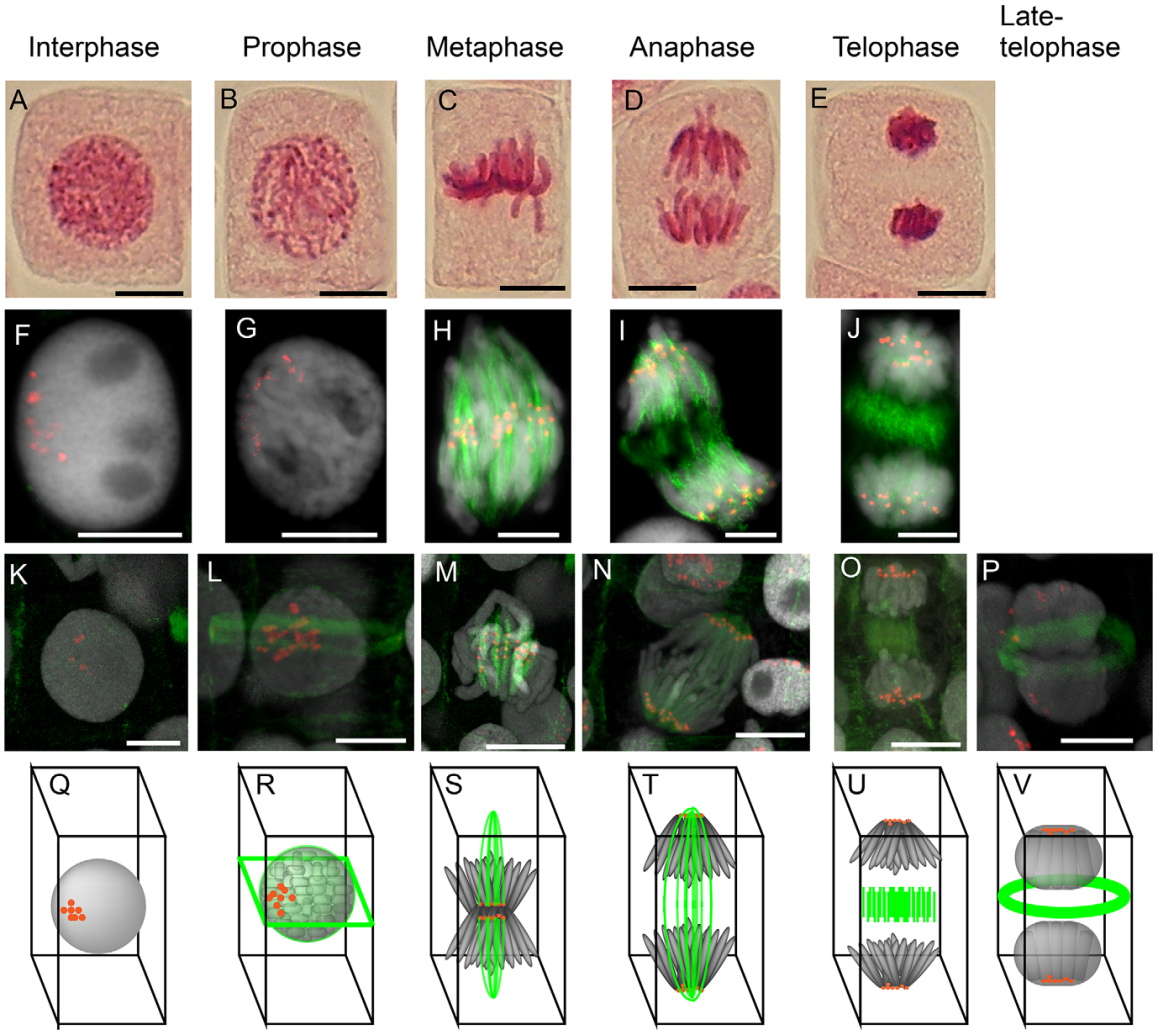
Comparison of acetic orcein staining, immunostaining and immunohistochemical staining in mitotic cells of onion. Chromosome pictures of acetic-orcein-staining (A–E), general immunostaining (F–J), and immunostaining of sections (K–P). Red, green and gray signals in F∼P are signals of anti-AfiCENH3 antibody, anti-α-tubulin antibody and DAPI, respectively. Scale bar, 10 µm. (G–V) A set of diagrams based on the results in K–P.

Additionally, 3D images constructed from sets of Z-stack pictures clearly revealed the 3D structure of nuclei and chromosomes and positioning of centromeres and α-tubulins during cell division (Figure 3G and S2). The positioning of centromeres and α-tubulin made it possible to distinguish the various phases of cell division. For example, at interphase, faint α-tubulin signals appeared on cell walls and CENH3 signals gathered on smooth nuclei (Figure 4K and Q, and Figure S3). From preprophase to prophase, PPB on cell walls were clearly visualized by the α-tubulin signals, and gathered CENH3 signals were observed on weakly condensed chromosomes (Figure 4L and R, and Figure S4). Faint α-tubulin signals were also observed on the nuclear membrane at prophase (Figure 4L and R, and Figure S5). Vertical microtubule signals and gathered centromeric signals at the equatorial plane were observed in metaphase cells (Figure 4M and S, and Figure S6). At anaphase, separated CENH3 signals on chromatids moved to each of the poles, and vertical microtubule signals remained visible (Figure 4N and T, and Figure S7). At telophase, the vertical microtubule signals disappeared, and α-tubulin signals were observed on the equatorial plane, although the CENH3 signals remained at the poles (Figure 4O and U, and Figure S8). The gathered α-tubulin signals adopted a ring-shaped appearance on the equatorial plane in late-telophase cells (Figure 4P and V, and Figure S9).

### DNA Sequences Coprecipitated with AfiCENH3

A ChIP cloning experiment was performed using the anti-AfiCENH3 antibody and chromatin extract from *A. fistulosum* leaves in an effort to identify centromeric DNA sequences in this species. DNA from the Pel fraction was extracted and then cloned into a plasmid vector. Slot-blot hybridization using extracted DNA from the Pel fraction as a probe showed strong signals in 21 of the 48 clones investigated (data not shown).

**Figure 5:**
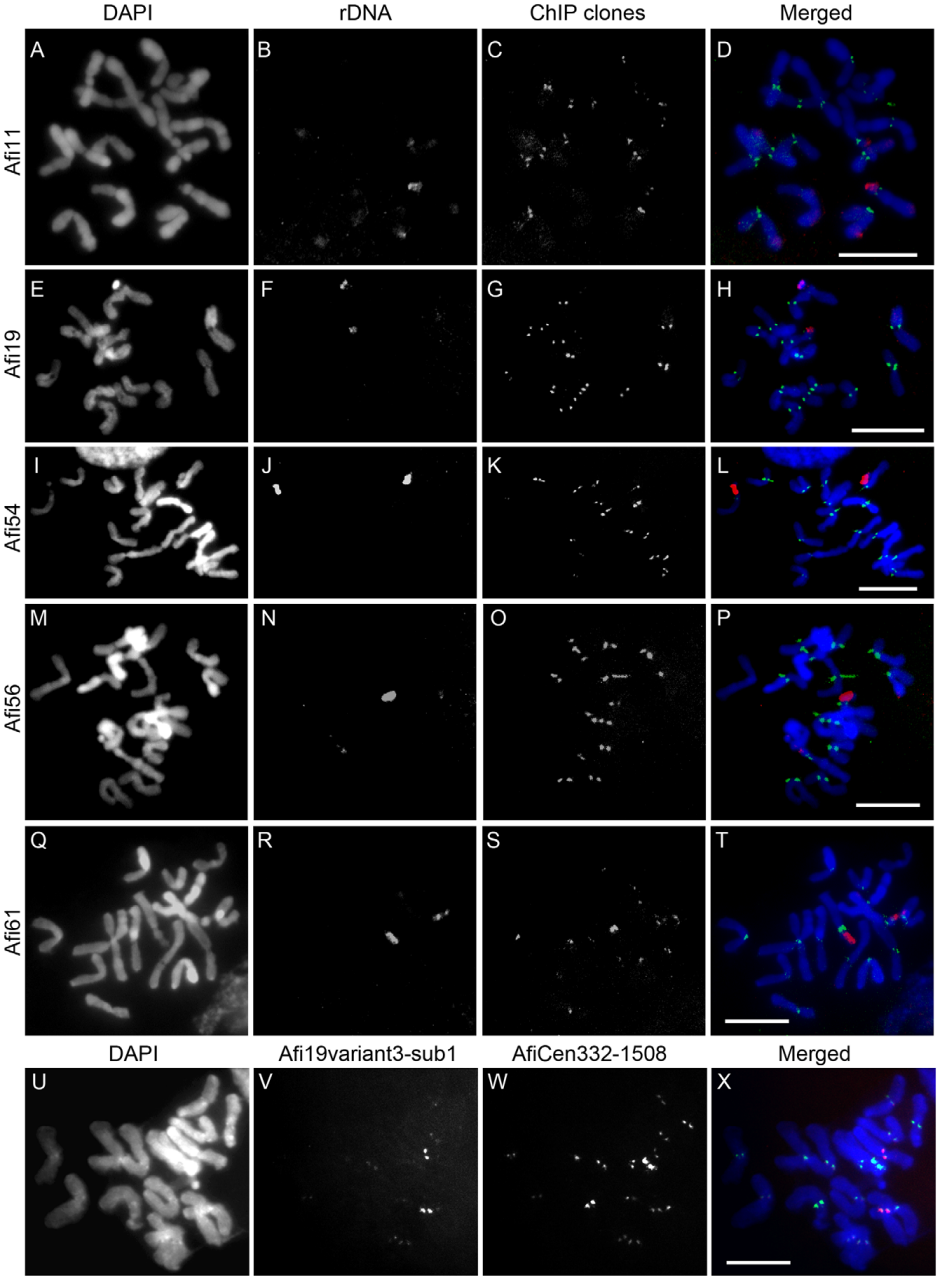
FISH using the ChIP clones and related sequences. (A), (E), (I), (M), (Q) and (U): DAPI stained *A. fistulosum* chromosomes. (B), (F), (J), (N) and (R): FISH signals of rDNA. (C): FISH signals of Afi11. (G): FISH signals of Afi19. (K): FISH signals of Afi54. (O): FISH signals of Afi56. (S): FISH signals of Afi61. (V): FISH signals of Afi19variant3-sub1 (W): FISH signals of AfiCen332-1508. (D): merged image of (A–C). (H): merged image of (E–G). (L): merged image of (I–K). (P): merged image of (M–O). (T): merged image of (Q–S). (X): merged image of (U–W). Scale bar, 10 µm.

To determine chromosomal localization, FISH analysis was performed using mitotic chromosome spreads of *A. fistulosum* and five of the 21 clones examined showed clear centromeric signals on the chromosomes (Figure 5). With these five clones, FISH signals appeared on the centromeres of all 16 chromosomes, although the intensities differed among the clones. One of the identified clones, Afi11 (GenBank accession number AB735740), showed 16 centromeric signals in *A. fistulosum*, although one pair was weaker than the others (Figure 5A–D). Afi19 (GenBank accession number AB735741) and Afi56 (GenBank accession number AB735743) also showed similar hybridization patterns as Afi11 (Figure 5E–H and M–P). For Afi54 (GenBank accession number AB735742), 12 strong and four weak signals were observed (Figure 5I–L). The remaining clone, Afi61 (GenBank accession number AB735744), showed one strong signal pair and 14 weak signals on the centromeres (Figure 5Q–T).

**Figure 6:**
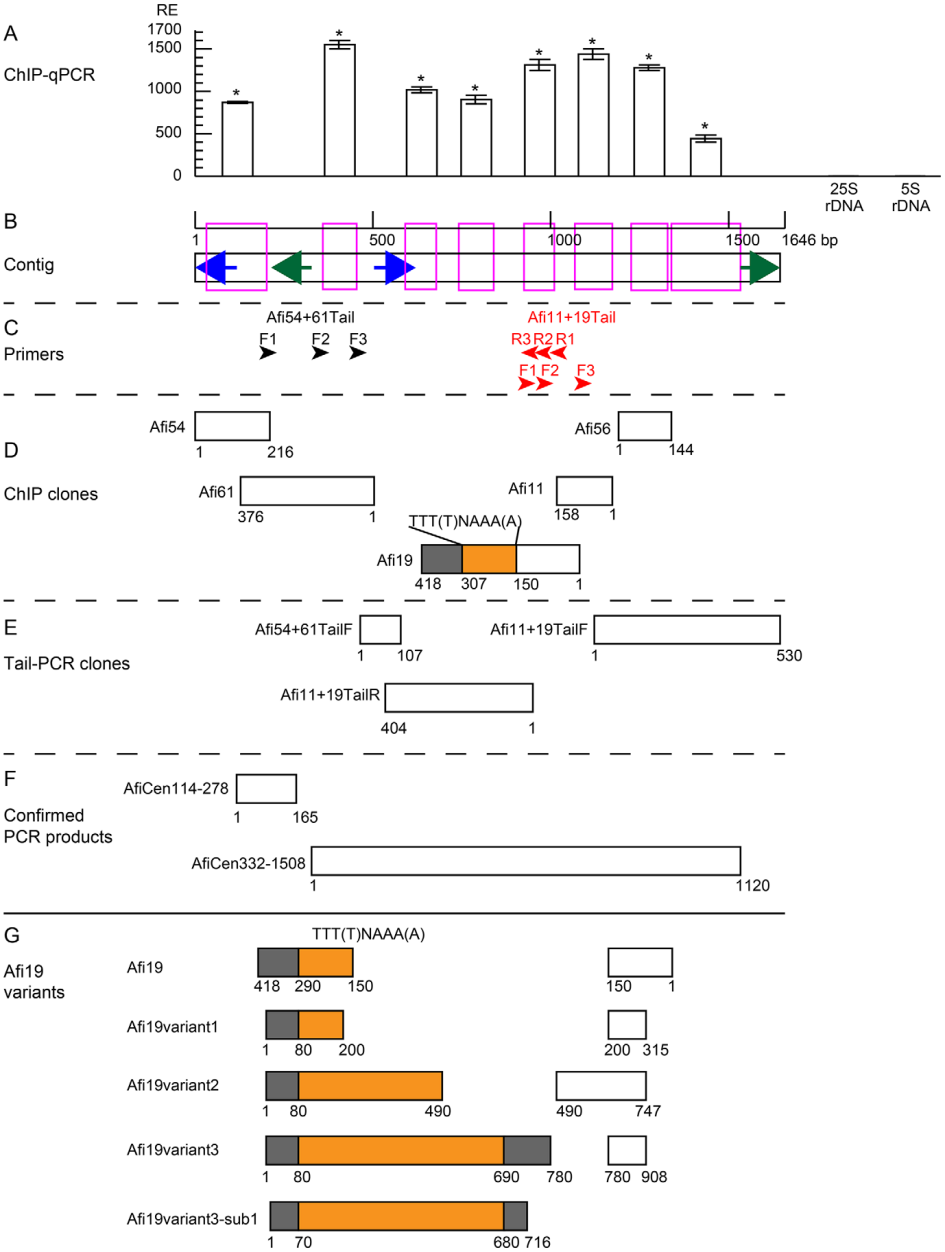
Sequence and qPCR analysis of centromeric DNA sequences of *A. fistulosum*. (A) ChIP-qPCR using anti-AfiCENH3 and chromatins from *A. fistulosum*. The columns and error bars represent the average relative enrichments (RE) and standard error from four independent ChIP experiments, respectively. Amplified regions of each qPCR are indicated by magenta boxes under the column in (B). 25SrDNA and 5SrDNA were used as non-centromeric controls. Statistical significance of differences between 25SrDNA and other sequences were tested using the Tukey’s Honesty Significant Difference test (*P<0.01). (B) A contig made by the ChIP (D) and Tail (E) clones. The blue and green arrows indicate the positions of two inverted repeats. (C) Arrow heads indicate primer position for the Tail-PCR. (D) Position of the ChIP clones. Regions containing non-homologous minisatellite and unique sequences in Afi19 are indicated by orange and gray boxes, respectively. (E) Position of the Tail-PCR clones. (F) Position of confirmed PCR products. (G) Afi19 and its variants. Regions containing non-homologous minisatellite and unique sequences in Afi19 are indicated by orange and gray boxes, respectively.

The DNA sequences of the aforementioned five clones were determined and used as queries in BLAST searches, but no sequence homology was found in GenBank. Comparison of the five sequences revealed a 32 bp homologous region between Afi11 and Afi19, and a 91 bp homologous region between Afi54 and Afi61 (Figure 6D).

In an effort to determine the overall structure of the Afi centromeric repeats, thermal asymmetric interlaced-PCR (Tail-PCR) was performed using primers designed based on the sequences of the Afi centromeric clones. As a result, Tail-PCR in combination with three specific primers (Afi54+61Tail-F1, F2 and F3) and three degenerate primers (D1-3) amplified a 107 bp fragment, Afi54+61TailF (GenBank accession number AB735745) (Table S2 and Figure 6E). Tail-PCR using Afi11+19Tail primer sets (F1-3 or R1-3) and degenerate primers amplified 530 bp and 404 bp fragments, Afi11+19TailF (GenBank accession number AB735746) and Afi11+19TailR (GenBank accession number AB735747), respectively (Figure 6E). Since the DNA sequence of Afi56 was found in Afi11+19TailF (Figure 6D and E), a 1646 bp contig was successfully generated (Figure 6B, D and E), although no sequence similarity was found in the database. It was noted that the region comprising base pair positions 151–418 of Afi19 has no sequence similarity to the contig, and part of this region (positions 151–307) contains degraded minisatellites comprising the unit sequence TTT(T)NAAA(A) (Figure 6D). To confirm the nature of the contig, a 30-cycle PCR experiment was performed using primer sets designed from the contig sequence and 200 ng *A. fistulosum* genomic DNA as a template. Under these PCR conditions, no single-copy sequences in the species are expected to be amplified, and only repetitive sequences should be amplified. The results showed that a region encompassing base pair positions 114–278 (AfiCen114-278) or 332-01508 (AfiCen332-1508) of the contig was amplified (Figure 6F), whereas no amplification was found between these two regions (data not shown). These results imply that an end of the AfiCEN repetitive units corresponds to the region comprising base pair positions 279–331 of the contig, and suggest that Afi61 contains a junction between the units. Additionally, two sets of inverted repeats were found at positions 1–124 and 501–624, and at 215–336 and 1511–1637 (Figure 6B).

Co-localization of the AfiCEN sequences and AfiCENH3 were investigated by qPCR using isolated DNA from the ChIP experiment and the anti-AfiCENH3 antibody (Figure 6A). All investigated regions in the contig showed higher RE (447.3–1549.2) than that of the non-centromeric control (25SrDNA: RE = 3.5 SE = 0.4 n = 4) and the other non-centromeric repeat (5SrDNA: RE = 4.1 SE = 0.3 n = 4), implying that all the regions colocalized with AfiCENH3 (Figure 6A). Actually, the AfiCen332–1508 PCR product showed stronger centromeric FISH signals than the original ChIP clones on the centromeric regions of *A. fistulosum* chromosomes (Figure 5U–X). FISH also supported their centromeric specificity.

Since the latter two thirds of the Afi19 sequence (positions 151–418) was not included in the AfiCEN contig, it was investigated by PCR and FISH. A set of primers, Afi19-F48 and -R404, was designed from the Afi19 sequence to determine any possible variation in the region. PCR using the primer yielded various products, three of which, Afi19variant1-3, were cloned and sequenced (Figure 6G). In the variants, the minisatellites and their flanking regions varied in copy number and DNA sequence, respectively (Figure 6G). To confirm centromeric specificity, the minisatellites and their franking regions were amplified by PCR using a set of primers, Afi19variant3-F6 and -R722, and cloned. When one of the clones, Afi19variant3-sub1, was used as a FISH probe, centromeric signals appeared on all *A. fistulosum* chromosomes (Figure 5U-X). This result implies that the minisatellites themselves are also centromeric.

## Discussion

In this study, we identified CENH3-coding cDNAs in four *Allium* species including *A. cepa*, *A. fistulosum*, *A. sativum* and *A. tuberosum*. The length of the amino acid sequences deduced from the cDNAs was identical, although some species-specific variations in sequence were found. The antibody raised against the N-terminal sequence of AfiCENH3 showed centromere-specific immunosignals in all *Allium* species used. Using the antibody, a ChIP assay and cloning was successful in identifying centromeric repeats in *A. fistulosum*.

*Allium* species have been used as cytogenetic material in biology classes due to the large size of the chromosomes and high mitotic indices in root-tips. However, information concerning cell division is limited in comparison with that of animal cells since the behavior of the centromeres has yet to be delineated, even in other plant species possessing large chromosomes [2]–[4]. In an effort to demonstrate the importance of visualizing centromeres during cell division, acetic-orcein-stained mitotic cells were compared with immunolabeled cells showing centromeres and spindle fibers (Figure 4). As shown in Figures 2–5, utilizing the results obtained when employing the antibody and FISH probes should enhance the utility of cytological textbooks, and assist in making such experiments a much more attractive proposition.

In some cases, anti-CENH3 antibodies could recognize CENH3 of closely related species. For example, anti-OsCENH3 antibody raised based on an amino acid sequence of rice CENH3 [15] recognized CENH3s of all Poaceae species investigated [17], [28], [29], [41]–[43]. This wide cross-reactivity of the antibody has been used to isolate centromeric DNA sequences from a number of Poaceae species. Similarly, anti-GmCENH3 antibody against soybean CENH3 also recognized CENH3 of *Astragalus sinicus*, and was utilized for isolation of the centromeric DNA [21], [22]. The anti-AfiCENH3 antibody raised also showed wide cross-reactivity for CENH3s in all Alliaceae species tested (Figure 2 and S1), indicating that the antibody is potentially useful for isolating centromeric DNA sequences from other Alliaceae species.

In some plant species, colocalization of the centromeric DNA and CENH3 has been confirmed using ChIP with anti-CENH3 antibodies [15], [17], [18], [21], [22], [24], [25], [28]–[31]. These confirmed centromeric DNA sequences could be grouped into two categories, minisatellite and retrotransposons. Although minisatellites are major components of animal and plant centromeres, retrotransposons are also major components in Poaceae species and tobacco [15], [17], [24], [28], [31], [44]. For example, centromeric retrotransposons are more abundant than centromeric minisatellite among maize chromosome 4, 6 and 9 [44]. In this study, centromeric DNA sequences of *A. fistulosum* were identified by ChIP and Tail-PCR experiments, although the entire structure and distribution pattern of these repeats remain unclear. We constructed a plasmid library containing 10-kb inserts on average, and screened the clones containing the repeats in the library (data not shown). However, we were unable to isolate a plasmid clone containing these repeats, suggesting that longer sequences containing these repeats might be unstable in the plasmid. In order to reveal the overall organization in the centromeres, further analyses using BAC libraries may be needed.

Since CENH3 have been characterized species limited in Poaceae and Juncaceae among monocot species, it has been difficult to evaluate specialty of CENH3 in holocentric species [15]–[17], [19], [26], [27]. In this study, we investigated CENH3s of Alliaceae species, and phylogenetic analyses using these sequences allowed us to identify two separate monocot clades (Figure 1B). These clades may have resulted from the different centromere types, monocentric and holocentric. Since information concerning CENH3 in holocentric species is limited in plants, further analyses would be needed to elucidate the special features of holocentric species.

## Materials and Methods

### Plant Material

Seeds of *A. fistulosum* (2*n* = 2*x* = 16) and *A. tuberosum* (2*n* = 4*x* = 32) and bulbs of *A. cepa* (2*n* = 2*x* = 16) and *A. sativum* (2*n* = 2*x* = 16) were obtained from commercial sources. Seeds were germinated on moistened filter paper at room temperature, and bulbs were germinated by hydroponic culture. Following germination, plants were transplanted to soil in pods and then grown in a greenhouse.

### BLAST Search of CENH3 Coding Genes

An EST sequence encoding onion CENH3 was identified from the gene index project database (http://compbio.dfci.harvard.edu/tgi/plant.html) using the tblastn program (http://compbio.dfci.harvard.edu/tgi/cgi-bin/tgi/Blast/index.cgi) and the amino acid sequence of OsCENH3 [15] as a query.

### RNA Isolation and PCR

One-week-old seedlings (*A. fistulosum* and *A. tuberosum*) and two-day-old roots (*A. cepa* and *A. sativum*) were used for RNA isolation. Total RNA was extracted using an RNeasy Plant Mini kit (Qiagen).

A full-length cDNA sequence of a CENH3-coding gene in *A. cepa* (AceCENH3) was isolated by RACE. For 3′RACE, the primer AceCENH3-3RACE (Table S1) was designed from a putative onion CENH3 sequence identified by the BLAST search, and used with a SMARTer RACE cDNA Amplification Kit (Clontech). Another primer, AceCENH3-5RACE (Table S1), was designed from the sequences determined by 3′RACE and used for 5′RACE.

Since sequences of CENH3-coding genes were unknown in other *Allium* species, parts of the sequences were amplified by PCR using cDNA from the isolated RNA. The cDNAs were synthesized from the total RNA using the ProtoScript First Strand cDNA Synthesis kit (New England Biolabs), and the cDNA and primers, AceCENH3-HFD-F and AceCENH3-HFD-R (Table S1), based on the AceCENH3 sequence were used for PCR to amplify fragments of CENH3 orthologs in the other *Allium* species. Based on sequences of amplified fragments in the PCR, primers (AfiCENH3-3RACE, AtuCENH3-3RACE, AsaCENH3-3RACE, AfiCENH3-5RACE, AtuCENH3-5RACE and AsaCENH3-5RACE in Table S1) were designed and used for RACE as in *A. cepa*.

### Sequencing and Sequence Alignment

Amplified PCR products were cloned into pGEM-T easy vector (Promega) and sequenced from both ends using a BigDye Terminator v1.1 cycle sequencing kit and an ABI PRISM 310 genetic analyzer (Applied Biosystems). Putative amino acid sequences were deduced from the DNA sequences determined, and aligned with those of CENH3 from other plant species and canonical histone H3 of rice using the Clustal X software program [45]. Phylogenetic relationships among the CENH3s were analyzed by the neighbor-joining method [46].

### Immunostaining

Based on the amino acid sequence deduced from the full-length cDNA of CENH3 from *A. fistulosum* (AfiCENH3), a peptide corresponding to the N-terminus of AfiCENH3 (H_2_N-ARTKQMAHKKLRRTLNVDEA-COOH) was synthesized and injected into two rabbits. The raised antisera were purified using an affinity sepharose column comprising the aforementioned peptide.

Immunostaining was conducted as previously described [16], [26] with minor modifications. Root tips of two-day-old *Allium* plants were fixed for 20 min in microtubules stabilizing buffer (50 mM PIPES, pH 6.9, 5 mM MgSO4 and 5 mM EGTA) containing 3% (w/v) paraformaldehyde and 0.2% (v/v) Triton X-100, and washed twice in phosphate-buffered saline (PBS). The fixed tips were digested for 1 h at 37°C with 1% (w/v) cellulase Onozuka RS (Yakult Pharmaceutical Industry Co. Ltd) and 0.5% (w/v) pectolyase Y-23 (Seishin Pharmaceuticals) mixture dissolved in PBS, washed twice in PBS, and then squashed onto slides coated with poly-L-lysine (Matsunami). A 1∶100 dilution of the purified anti-AfiCENH3 antibody and a 1∶100 dilution of mouse monoclonal anti-α-tubulin antibody (Sigma) were applied to the slides. After washing in PBS, the antibodies were detected using a 1∶100 dilution of Alexa Fluor 555-labeled anti-rabbit antibodies (Molecular Probes) and a 1∶100 dilution of Alexa Fluor 488-labeled anti-mouse antibodies (Molecular Probes), respectively. Chromosomes were counterstained with 0.1 µg/ml 4,6-diamino-2-phenylindole (DAPI). Immunosignals and stained chromosomes were captured using a chilled charge-coupled device (CCD) camera (AxioCam HR, Carl Zeiss) and images were pseudo-colored and processed using the AxioVision software (Carl Zeiss).

### Immunohistochemical Staining of Root Slices

Immunohistochemical staining was conducted as described by Yamaji and Ma (2007) [47] with minor modifications. Onion roots of a two-day-old hydroponic culture were fixed and washed as described above for the immunostaining. The fixed roots were embedded in 5% agar and sectioned 100-µm thick using a microslicer (LinearSlicer PRO10; Dosaka EM) for horizontal (round) slices. In the case of vertical slices, fixed roots were sectioned without the embedding step. These sections were transferred onto slide glasses, and then macerated with 0.1% (w/v) pectolyase Y-23 and 0.3% (w/v) Triton X-100 in PBS at room temperature. The macerated slices were washed three times in PBS, and blocked with 5% (w/v) bovine serum albumin (BSA) in PBS. 1∶100 dilutions of the purified anti-AfiCENH3 antibody and monoclonal anti-α-tubulin antibody produced in mouse were applied to the slides. After washing in PBS, the slices were blocked again under the same conditions. Blocked samples were then exposed to 1∶1000 diluted secondary antibodies, Alexa Fluor 555-labeled anti-rabbit antibodies and Alexa Fluor 488-labeled anti-mouse antibodies, and nuclei and chromosomes were counterstained by DAPI. Immunosignals and stained chromosomes were observed with a laser-scanning confocal microscope (LSM700; Carl Zeiss). Obtained data were analyzed using Zeiss LSM Image Browser software (Carl Zeiss).

### ChIP Cloning

ChIP was performed as previously described [31] with minor modifications, using leaves of adult plants of *A. fistulosum* and the anti-AfiCENH3 antibody. Leaves were ground in liquid nitrogen using a mortar and a pestle and then suspended in TBS buffer (1 mM Tris-HCl, pH 7.5, 3 mM CaCl_2_ and 2 mM MgCl_2_) containing 0.1 mM phenylmethylsulfonyl fluoride (PMSF) and protease inhibitor cocktail (Roche). The suspension was filtered through Miracloth (Calbiochem) and nuclei were collected by centrifugation (600×g, 10 min). Nuclei were digested with micrococcal nuclease (Sigma) to produce a chromatin solution. Following overnight incubation with the antibody at 4°C, the chromatin solution was separated into Sup (unbound) and Pel (bound) fractions using Dynabeads Protein A (Invitrogen). For mock experiments, only the beads were incubated in the chromatin solution. DNA was purified from the Pel fractions by phenol/chloroform extraction followed by ethanol precipitation.

Part of the purified DNA was blunted with T4 DNA polymerase (Toyobo). The blunted DNA was treated with Taq polymerase (Promega) to append an adenine residue at the 3′-ends, and then cloned into pGEM-T easy vector (Promega).

### Slot Blot Hybridization

DNA cloned from the Pel fraction was blotted onto a nylon membrane (Biodyne Plus, Pall) using a slot-blotter. The Pel DNA was labeled using a DIG High prime labeling kit (Roche) to screen high-copy sequences preferentially present in the Pel pool. The blotted DNAs were hybridized with the DIG-labeled probe, and hybridized probes were detected using a DIG Luminescent Detection Kit (Roche). Luminescent signals were captured and quantified using the LAS1000 plus system (Fuji film).

### Fluorescence *in situ* Hybridization

Root tips of *A. fistulosum* were pretreated at 0°C for 20 h in water. Pretreated root tips were fixed in ethanol/glacial acetic acid (3∶1), and then dividing cells in the root tips were squeezed in 45% acetic acid on slide glasses. For probe DNA, inserts of plasmid clones or PCR products were labeled using a Dig-Nick Translation Mix (Roche) or a Biotin-Nick Translation Mix (Roche). To detect nucleolar organizer regions, an 18S-5.8S-28SrDNA clone from wheat (pTa71) was used [48]. The FISH procedure employed was essentially that of Mukai et al. [49] with some modifications. The DIG- and biotin-labeled probes were visualized using rhodamine-conjugated anti-digoxigenin antibody (Roche) and Fluorescein-conjugated avidin DN (Vector laboratories), respectively. Chromosomes were counterstained as described above for the immunostaining. Fluorescent signals were captured using a fluorescence microscope (Zeiss Axioskop) coupled with a CCD camera (model 4880, Hamamatsu Photonics). The FISH images were pseudo-colored and merged using Photoshop 5.0 software (Adobe).

### Tail-PCR Analysis

Tail-PCR analyses were conducted as previously described [50] with minor modifications. Genomic DNA was isolated from leaves of adult *A. fistulosum* using a DNeasy Plant Mini kit (Qiagen). The reactions were conducted with specific primer sets (Afi11+19Tail-F1-3, Afi11+19Tail-R1-3 and Afi54+61Tail-F1-3 in Table S2) and three degenerate primers (D1-3 in Table S2).

### Real-time Quantitative PCR Analysis

qPCR analyses were conducted on a 7500 Real Time PCR System (Applied Biosystems) using a SYBR Premix Ex Taq kit (Takara). A set of PCR primers (25SrDNA-F and 25SrDNA-R in Table S3) was designed to amplify 25S rDNA for use as a non-centromeric control. In addition to the PCR primers, nine primer sets were designed to amplify 5SrDNA (Afi5SrDNA-F and Afi5SrDNA-R) and specific regions of the *A. fistulosum* centromeric sequences determined by the ChIP or Tail-PCR experiments (Table S3). The qPCR comprised 40 cycles of 94°C for 5 s and 50°C for 34 s, and a set of dissociation steps were conducted at 95°C for 15 s, 60°C for 30 s, and 95°C for 15 s to verify amplified products. Purified Pel DNA from four independent ChIP reactions using anti-AfiCENH3 and the mocks were used for the qPCR. Pel DNA from the mock was used as a negative control. PCR products were quantified using standard curves made by a dilution set of the genomic DNA as templates. Relative enrichment was calculated by the following formula: relative enrichment = amount of the sequence in Pel from the antibody fraction/amount of the sequence in Pel from the mock fraction. The probability that the extra-centromeric control and other repeats belong to the same group was determined by analysis of variance (ANOVA) using the ezANOVA software. Pairwise comparisons of each group were conducted by the Tukey’s Honestly Significant Difference (Tukey HSD) test using the ezANOVA software.

## Supporting Information

Figure S1 **Immunostaining of chromosomes of** ***Allium*** **species using an anti-AfiCENH3 antibody.** (A), (D), (G) and (J): DAPI stained chromosomes. (B), (E), (H) and (K): Immunosignals of an anti-AfiCENH3 antibody. (C): Merged image of (A and B). (F): Merged image of (D and E). (I): Merged image of (G and H). (L): Merged image of (J and K). (A–C): *A. fistulosum.* (D–F): *A. cepa.* (G–I): *A. sativum*. (J–L): *A. tuberosum.* Scale bar, 10 µm.
(TIF)

Figure S2 **A 3D image constructed from a set of Z-stack pictures of a root slice.** Anti-AfiCENH3, anti-α-tubulin and DAPI signals are indicated in red, green and gray, respectively. Scale bar, 100 µm.
(TIF)

Figure S3 **A 3D video including interphase cells.** Anti-AfiCENH3, anti-α-tubulin and DAPI signals are indicated in red, green and gray, respectively.
(M4V)

Figure S4 **A 3D video including a prophase cell.** Anti-AfiCENH3, anti-α-tubulin and DAPI signals are indicated in red, green and gray, respectively.
(M4V)

Figure S5 **A 3D image constructed from a set of Z-stack pictures of a prophase cell.** Anti-AfiCENH3, anti-α-tubulin and DAPI signals are indicated in red, green and gray in (A), respectively. The anti-α-tubulin signals in (A) are shown in gray in (B). Scale bar, 10 µm.
(TIF)

Figure S6 **A 3D video including a metaphase cell.** Anti-AfiCENH3, anti-α-tubulin and DAPI signals are indicated in red, green and gray, respectively.
(M4V)

Figure S7 **A 3D video including an anaphase cell.** Anti-AfiCENH3, anti-α-tubulin and DAPI signals are indicated in red, green and gray, respectively.
(M4V)

Figure S8 **A 3D video including a telophase cell.** Anti-AfiCENH3, anti-α-tubulin and DAPI signals are indicated in red, green and gray, respectively.
(M4V)

Figure S9 **A 3D video including a late-telophase cell.** Anti-AfiCENH3, anti-α-tubulin and DAPI signals are indicated in red, green and gray, respectively.
(M4V)

Table S1 **Primers used for RACE and RT-PCR.**
(DOC)

Table S2 **Primers used for Tail-PCR.**
(DOC)

Table S3 **Primers used for qPCR.**
(DOC)
