# Supplementary figures and images for: Chromosome Dynamics Visualized with an Anti-Centromeric Histone H3 Antibody in Allium
Source: PLoS One. 2012 Dec 7;7(12):e51315. doi: 10.1371/journal.pone.0051315 (PMC3517398; doi:10.1371/journal.pone.0051315)

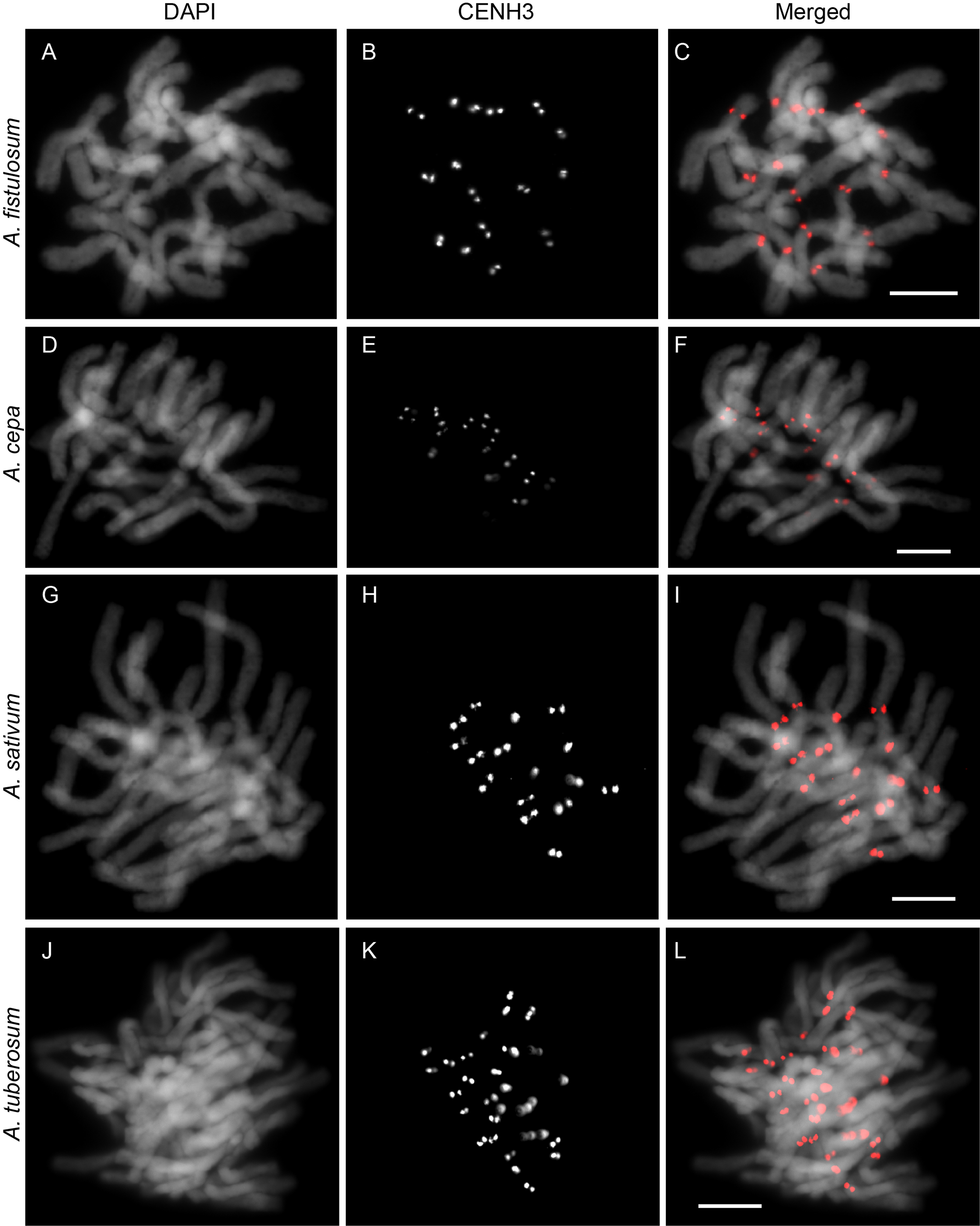

Supplement: Figure S1 — Immunostaining of chromosomes of Allium species using an anti-AfiCENH3 antibody. (A), (D), (G) and (J): DAPI stained chromosomes. (B), (E), (H) and (K): Immunosignals of an anti-AfiCENH3 antibody. (C): Merged image of (A and B). (F): Merged image of (D and E). (I): Merged image of (G and H). (L): Merged image of (J and K). (A–C): A. fistulosum. (D–F): A. cepa. (G–I): A. sativum. (J–L): A. tuberosum. Scale bar, 10 µm. (TIF) [file pone.0051315.s001.tif]

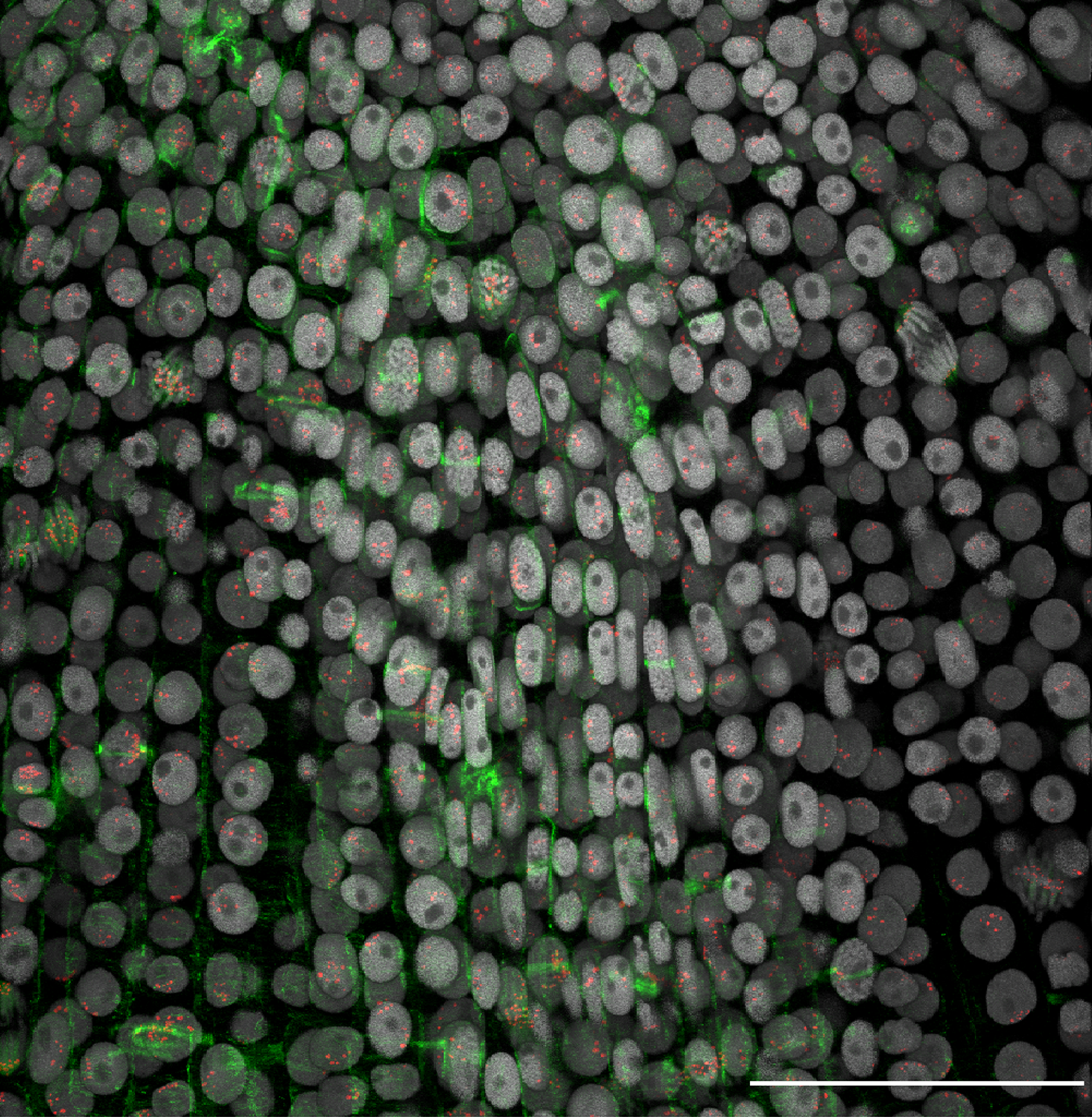

Supplement: Figure S2 — A 3D image constructed from a set of Z-stack pictures of a root slice. Anti-AfiCENH3, anti-α-tubulin and DAPI signals are indicated in red, green and gray, respectively. Scale bar, 100 µm. (TIF) [file pone.0051315.s002.tif]

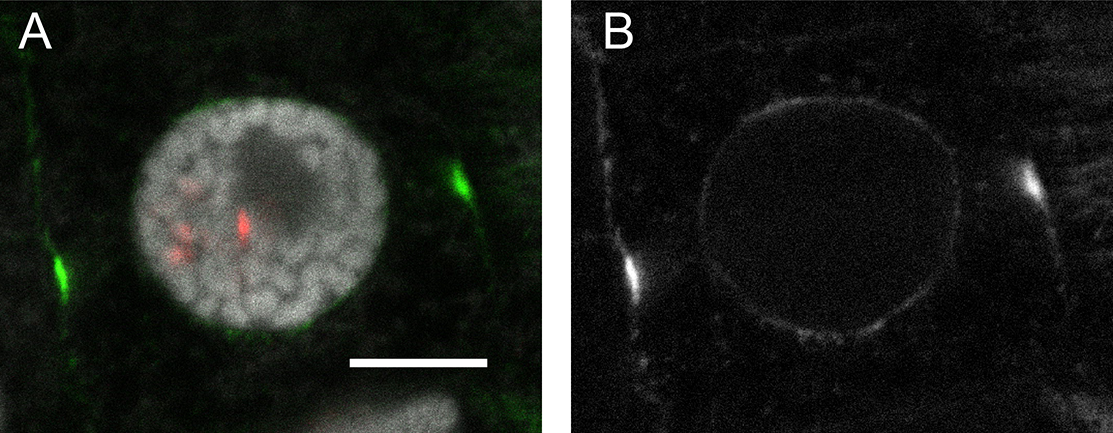

Supplement: Figure S5 — A 3D image constructed from a set of Z-stack pictures of a prophase cell. Anti-AfiCENH3, anti-α-tubulin and DAPI signals are indicated in red, green and gray in (A), respectively. The anti-α-tubulin signals in (A) are shown in gray in (B). Scale bar, 10 µm. (TIF) [file pone.0051315.s005.tif]
