# Supplementary material for: Chromosome Dynamics Visualized with an Anti-Centromeric Histone H3 Antibody in Allium
Source: PLoS One. 2012 Dec 7;7(12):e51315. doi: 10.1371/journal.pone.0051315 (PMC3517398; doi:10.1371/journal.pone.0051315)
Supplement: Table S1 — Primers used for RACE and RT-PCR. (DOC) [file pone.0051315.s010.doc]

**Table S1. Primers used for RACE and RT-PCR**

| Name | Sequence |
| --- | --- |
| AceCENH3-3RACE | 5’-TCCTTCTTTGCCCCACTTTCTCC-3’ |
| AceCENH3-5RACE | 5’-CAGCTGTATATCCTTTCGCATCA-3’ |
| AceCENH3-HFD-F | 5’-TGCTCCATTTATCAGGCTTGTG-3’ |
| AceCENH3-HFD-R | 5’-AAATGTCTAGCACCACCAATCC-3’ |
| AfiCENH3-3RACE | 5’-GCGTTACACTGATGCGAAAGGAT-3’ |
| AfiCENH3-5RACE | 5’-CAGCTGTATATCCTTTCGCATCA-3’ |
| AtuCENH3-3RACE | 5’-CGAAGCGCGTTACAGTGATGCAA-3’ |
| AtuCENH3-5RACE | 5’-CAGCTGTATATCCTTTCGCATCA-3’ |
| AsaCENH3-3RACE | 5’-CGAAGCGCGTTACAGTGATGCAA-3’ |
| AsaCENH3-5RACE | 5’-CTCGCTAGCTGAATGTCCCTTTG-3’ |
