# Supplementary material for: Chromosome Dynamics Visualized with an Anti-Centromeric Histone H3 Antibody in Allium
Source: PLoS One. 2012 Dec 7;7(12):e51315. doi: 10.1371/journal.pone.0051315 (PMC3517398; doi:10.1371/journal.pone.0051315)
Supplement: Table S2 — Primers used for Tail-PCR. (DOC) [file pone.0051315.s011.doc]

**Table S2. Primers used for Tail-PCR**

| Name | Sequence |
| --- | --- |
| D1 | 5’-NTCGASTWTSGWGTT-3’ |
| D2 | 5’-NGTCGASWGANAWGAA-3’ |
| D3 | 5’-WGTGNAGWANCANAGA-3’ |
| Afi54+61Tail-F1 | 5’-TAGATAAGCACAACAAAATGGCACGAG-3’ |
| Afi54+61Tail-F2 | 5’-AGCCTCTAGCCTCACTTTCTTTC-3’ |
| Afi54+61Tail-F3 | 5’-TCTCTTCGATTTGGGACCTTAGT-3’ |
| Afi11+19Tail-F1 | 5’-TTCAAAAACTAAGCCGGCTCTATACCAAATC-3’ |
| Afi11+19Tail-F2 | 5’-CCGTTTTAGGCATGCGTTACCC-3’ |
| Afi11+19Tail-F3 | 5’-CACACTCAGGAAAGCAGGCACGAACC-3’ |
| Afi11+19Tail-R1 | 5’-ACGGCAATATTTATAAGTTACATCAGG-3’ |
| Afi11+19Tail-R2 | 5’-GATTTGGTATAGAGCCGGCTTGGTTTTTGA-3’ |
| Afi11+19Tail-R3 | 5’-TAACGGGGAGAATCTAATACCTTGAAAAATC-3’ |
