# Supplementary material for: Chromosome Dynamics Visualized with an Anti-Centromeric Histone H3 Antibody in Allium
Source: PLoS One. 2012 Dec 7;7(12):e51315. doi: 10.1371/journal.pone.0051315 (PMC3517398; doi:10.1371/journal.pone.0051315)
Supplement: Table S3 — Primers used for qPCR. (DOC) [file pone.0051315.s012.doc]

**Table S3. Primers used for qPCR**

| Name | Sequence |
| --- | --- |
| 25SrDNA-F | 5’-GCGGGTAAACGGCGGGAGTAA-3’ |
| 25SrDNA-R | 5’-GACAGGGACAGTGGGAATCT-3’ |
| Afi5SrDNA-F | 5’-CGTGCTTGGGCGAGAGTAGTA-3’ |
| Afi5SrDNA-R | 5’-cgaatataaaaacggatggaagg-3’ |
| AfiCen-F35 | 5’-GCGTATCCCACTTTCCATT-3’ |
| AfiCen-R208 | 5’-TATATGAATTTGGACTGC-3’ |
| AfiCen-F364 | 5’-AGCCTCTAGCCTCACTTTCTTT-3’ |
| AfiCen-R450 | 5’-AATGCTTAGGGGTCACAGGT-3’ |
| AfiCen-F736 | 5’-TCAAATCAGAAAAACGAGACG-3’ |
| AfiCen-R829 | 5’-TAAAAAGAAGGATGGGTAAAATG-3’ |
| AfiCen-F889 | 5’-ATTTTGACTATTTGATTTTTC-3’ |
| AfiCen-R997 | 5’-TGATTTTGGTTTACGATTTG-3’ |
| AfiCen-F1053 | 5’-ATACACATTGCGGCATAGACTC-3’ |
| AfiCen-R1147 | 5’-ATCTATAAATTTAAAGGTTCAT-3’ |
| AfiCen-F1218 | 5’-AATTCTTGGGTTACTCC-3’ |
| AfiCen-R1310 | 5’-ATCAAAAGCAAAGAAATC-3’ |
| AfiCen-F1323 | 5’-GTAATACACAATCATAACAA-3’ |
| AfiCen-R1508 | 5’-TTCCAAGAGATCTAATACAT-3’ |
